# The Immunology of a Healing Response in Cutaneous Leishmaniasis Treated with Localized Heat or Systemic Antimonial Therapy

**DOI:** 10.1371/journal.pntd.0004178

**Published:** 2015-10-20

**Authors:** Ines Lakhal-Naouar, Bonnie M. Slike, Naomi E. Aronson, Mary A. Marovich

**Affiliations:** 1 Department of Medicine, Uniformed Services University of the Health Sciences, Bethesda, Maryland, United States of America; 2 Henry M. Jackson Foundation for the Advancement of Military Medicine, Bethesda, Maryland, United States of America; 3 U.S. Military HIV Research Program, Walter Reed Army Institute of Research, Silver Spring, Maryland, United States of America; 4 Walter Reed National Military Medical Center, Bethesda, Maryland, United States of America; The Ohio State University, UNITED STATES

## Abstract

**Background:**

The effectiveness of systemic antimonial (sodium stibogluconate, Pentostam, SSG) treatment versus local heat therapy (Thermomed) for cutaneous leishmaniasis was studied previously and showed similar healing rates. We hypothesized that different curative immune responses might develop with systemic and local treatment modalities.

**Methods:**

We studied the peripheral blood immune cells in a cohort of 54 cutaneous *Leishmania major* subjects treated with SSG or TM. Multiparameter flow cytometry, lymphoproliferative assays and cytokine production were analyzed in order to investigate the differences in the immune responses of subjects before, on and after treatment.

**Results:**

Healing cutaneous leishmaniasis lead to a significant decline in circulating T cells and NKT-like cells, accompanied by an expansion in NK cells, regardless of treatment modality. Functional changes involved decreased antigen specific CD4^+^ T cell proliferation (hyporesponsiveness) seen with CD8^+^ T cell depletion. Moreover, the healing (or healed) state was characterized by fewer circulating regulatory T cells, reduced IFN-γ production and an overall contraction in polyfunctional CD4^+^ T cells.

**Conclusion:**

Healing from cutaneous *Leishmaniasis* is a dynamic process that alters circulating lymphocyte populations and subsets of T, NK and NKT-like cells. Immunology of healing, through local or systemic treatments, culminated in similar changes in frequency, quality, and antigen specific responsiveness with immunomodulation possibly via a CD8^+^ T cell dependent mechanism. Understanding the evolving immunologic changes during healing of human leishmaniasis informs protective immune mechanisms.

## Author Summary

Globally, leishmaniasis treatment relies on the use of antimonial drugs (i.e. SSG). In an earlier study we showed that skin lesions due to *L*. *major* treated by the ThermoMed (TM) device healed at a similar rate and with less associated systemic toxicity than lesions treated with intravenous SSG. The current study compared the immune responses of these two therapeutic groups before, during and after therapy which may be relevant to resistance to reinfection and also in consideration for the development of local (versus systemic) therapy. Antimonials have immune effects on both the host and parasite while heat treatment locally kills the parasite and induces inflammation from a secondary burn. We demonstrated that healing from cutaneous *leishmaniasis* is a dynamic process associated with a modulation of immune responses independent of treatment modalities.

## Introduction

Leishmaniasis, a vector-borne parasitic disease, remains a pressing global health concern with 12 million persons infected, 2 million new infections each year, limited therapeutic options and no effective vaccine [1].

Healing cutaneous leishmaniasis (CL) relies on the development of an effective and balanced protective immune response. The intracellular parasite needs to be contained, while the pathologic immune response needs to be controlled. The murine model for *L*. *major* substantially contributed to our understanding of protective immunity and helped establish the T helper 1 (Th1)/Th2 paradigm that explained resistance and susceptibility to *Leishmania* infection [2,3]. This model demonstrated that T lymphocytes are key for the generation of this protective response through their IFN-γ production which activates macrophages to produce toxic nitrogen and oxygen metabolites to kill the intracellular amastigotes [4]. The Th1 cytokine profile, i.e. IFN-γ, TNF-α and IL-12, is crucial to eliminate *Leishmania* [5], while the development of a Th2 immune response with the production of IL-4, TGF-β and IL-10 favors parasite multiplication and fails to control the infection [6]. The quality of a T cell response, defined by the pattern of cytokine production at the single cell level, underscores the importance of polyfunctional CD4^+^T cells specifically producing IFN-γ, TNF-α and IL-2 for protection [7,8]. Additionally, immunoregulatory mechanisms involving regulatory and memory T cells can significantly influence leishmaniasis outcome [9].

The precise role of human CD4^+^T cell subsets, their cytokine patterns and the immune response pathways engaged during and after effective leishmaniasis therapy are incompletely understood. While pentavalent antimonial drugs (i.e. SSG, meglumine antimoniate) have been used to treat CL for decades [10], they are toxic, require extended duration of treatment, and drug resistant parasites have emerged as a problem [11,12]. The mechanism of action of SSG includes effects on both the host macrophage and parasite [13]. Thermotherapy is an alternative treatment for CL [14,15], delivering localized radiofrequency waves into skin lesions to physically destroy the temperature sensitive parasites. Thermomed (TM, Thermosurgery Technologies, Phoenix AZ), cleared by the Food and Drug Administration, is one of the most studied devices in use [15]. Clinical trials comparing local heat to systemic antimonial therapy showed similar CL cure rates [14,16–20].

We previously reported that subjects treated with the TM device showed similar healing by 2 and 12 months follow-up, with less associated systemic toxicity than those treated with intravenous SSG [21]. We hypothesized that an immunomodulatory systemic therapy would induce a different immune response compared to a locally applied physical treatment, though both methods were ultimately curative. This work comparatively evaluated the immune response profile over time in the participants treated with SSG or TM. We showed a modulation of immune response occurs during healing from cutaneous *leishmaniasis* independent of either treatment modality.

## Materials and Methods

### Ethics statement

All participants provided written informed consent and study protocols were approved by Institutional Review Boards at both WRAMC and the Walter Reed Army Institute of Research.

### Study population

All participants were U.S. military personnel referred to the Walter Reed Army Medical Center (WRAMC) for treatment of parasitologically confirmed *L*. *major* infection (Table 1). Details of the clinical trial are published [21]. Seven healthy uninfected control subjects were recruited under a separate protocol.

**Table 1:** Demographic characteristics and outcome presented by treatment arm.

Characteristic	SSG (n = 20)	TM (n = 19)	p value
**Median age in years (range)**	24.5 (18–57)	25 (20–41)	0.204*
**Male gender (%)**	95	100	1**
**Race (%)**			0.85***
** White**	65	57	
** Black**	15	26.3	
** Hispanic**	15	10.5	
** Filipino/Asian**	5	5.2	
**Median number of lesions**	3 (1–17)	3 (1–14)	0.99*
**Total area of lesions (mm)**	531.5 (100–3230)	691.5 (81–2533)	0.95*
**Median time since onset (days)**	137.5	145.5	0.7*
**Number healed at 6 months (%)**	18/20	15/19	0.407**

* Mann-Whitney

** Fisher exact

***Vassarstats

### Sample collection and storage

Whole blood subjects were drawn at time points designated “pre-treatment” (PRE), “on-treatment” (ON) and “post-treatment” (POST) (Days 0, 9±1 and 219±68 following treatment initiation, respectively). For pre- and on-treatment subjects, blood was drawn at WRAMC and processed fresh. At POST, blood was drawn at alternate medical facilities and shipped via overnight carrier for processing. Peripheral blood mononuclear cells (PBMC) were isolated from whole blood as previously described [22].

### Antibodies for flow cytometry

The following fluorescence-conjugated antibodies were used for multiparameter flow cytometry: CD3 (SK7), CD4 (SK3), CD8 (SK1), CD14 (M5E2), CD19 (HIB19) CD25 (2A3), IL-10 (JES3-19F1), TNF-α (Mab11), IL-2 (5344.111), γδ TCR (B1) (BD Biosciences, San Jose, CA); CD4 (SFCI12T4D11) (Beckman Coulter, Fullerton, CA); IL-17 (eBio64DEC17) and αβ TCR (IP26) (BioLegend, San Diego, CA); IFN-γ (4S.B3) (eBioscience, San Diego, CA). All antibodies were titrated prior to use to determine optimal staining concentrations. Flow cytometry data was acquired either on a FACS Calibur or LSR-II flow cytometer (BD Biosciences) and data analyzed using FlowJo software (TreeStar, Ashland OR).

### Fresh cell population phenotyping

Prior to cryopreservation, a PBMC aliquot was stained for cell surface markers and analyzed by flow cytometry. Markers included the BD SimulTEST (CD45, CD14) and BD MultiTEST (CD3, CD16, CD56, CD45, CD19) reagents. T cell populations were further analyzed by staining with CD3, CD4, CD8, and CD25. Following staining, cells were fixed in 2% paraformaldehyde, data collected with a FACS Calibur flow cytometer (BD Biosciences) and analyzed using FlowJo software (TreeStar, Ashland OR).

### Lymphocyte proliferation assay (LPA) and quantitation of secreted cytokines

Cryopreserved PBMC were thawed in complete media. A portion of the PBMC was depleted of CD8^+^T cells (CD8depl PBMC) using the Dynal CD8 Positive Isolation Kit (Invitrogen, Carlsbad CA). Total PBMC or CD8depl PBMC were plated in the presence of soluble *Leishmania* antigens from *L*. *major* parasites (SLA, 2.5 μg/mL, generous gift of Dr. Frank Neva) for 6 days at 37°C, 5% CO_2_. Pokeweed mitogen (PWM, 5 μg/mL, Sigma) was used as a positive control. Cell-free supernatant was collected from each well, triplicate subjects pooled, and used to quantify cytokines using the Q-Plex Human Cytokine–IR Array (Quansys Biosciences, Logan, UT) according to manufacturer’s protocol [23]. For LPA, cells were pulsed as previously reported [24].

### CFDA-SE labeling of PBMC

Cryopreserved PBMC were thawed and labeled with carboxyfluorescein diacetate succinimidyl ester (CFDA-SE, Invitrogen, Carlsbad, CA) according to the manufacturer’s instructions [25].

### Intracellular cytokine staining

Cryopreserved PBMC were thawed and incubated overnight at 37°C, 5% CO_2_. Cells were plated at 1 x 10^6^ per well and stimulated with *L*. *major* whole lysate (1μg/mL, generous gift from David Sacks) for 24 hours at 37°C, 5% CO_2_. Brefeldin A (10μg/mL, Sigma) was added to all wells at 18 hours. All cells were costimulated with 1 μg/mL CD28 and CD49d antibodies (BD Biosciences). Following stimulation, cells were stained for population identification markers (CD3, CD4, CD8, CD14 and CD19) and intracellular cytokine expression (TNF-α, IFN-γ, and IL-2). T cell receptor (TCR) phenotyping antibodies were included for the αβ TCR and γδ TCR.

### Statistics

All statistics were performed using GraphPad Prism 4.0 (GraphPad Software, San Diego, CA).

## Results

### Sample cohort description

Fifty-four U.S soldiers (98% male) with CL were enrolled and randomized to either local heat therapy (TM) or 10 days of intravenous SSG (Table 1). To evaluate the immune response profiles in these subjects, PBMC were isolated from whole blood at three time points. Pre-treatment (PRE) cells were collected upon enrollment into the study (n = 54, 100%). The on-treatment cells were collected on 9±1 treatment day (n = 54, 100%), and post-treatment (POST) subjects collected at a mean of 7 months (range 4.7–9.2 months), after treatment (n = 39, 72%). Because 39/54 participants provided cells at all time points, the majority of our analysis is restricted to this subcohort (Table 1). No significant differences were noted between treatment arms or subcohort and cohort regarding demographic characteristics, disease burden and therapy outcome.

### Lymphocyte populations differ between disease and healed state

**Fig 1:**
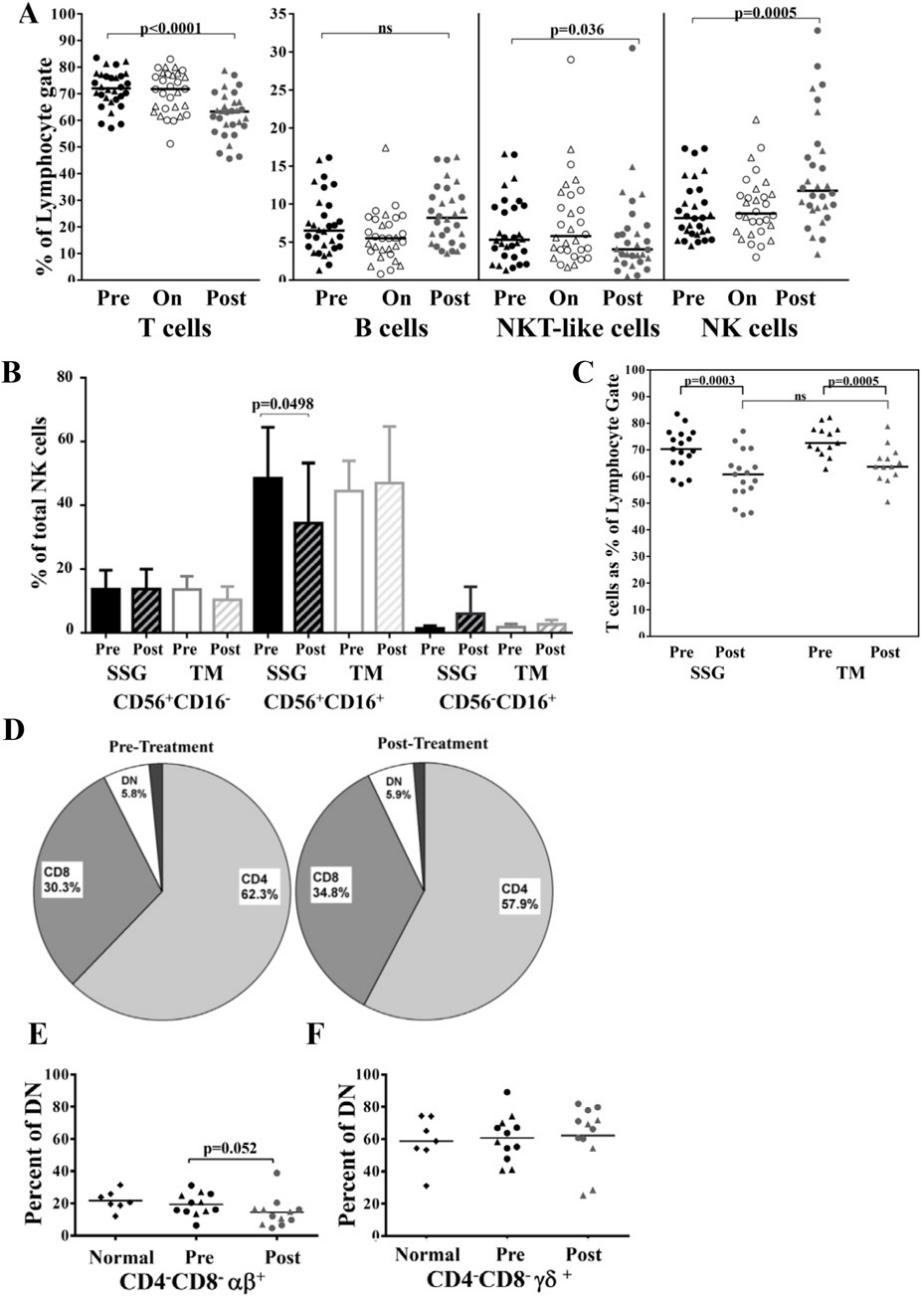
Characterization of lymphocyte populations by flow cytometry. Data is presented from 30 subjects (17 in the SSG arm and 13 in TM arm represented in circles and triangles respectively) for which cells from all three time points were available. **(A)** Percentage of lymphocytes positive for surface expression of CD3 (T cells), CD19 (B cells), CD16/CD56 (CD3^-^: NK cells; CD3^+^: NKT-like cells). **(B)** Distribution of subpopulations of NK cells based on CD16 and CD56 expression. **(C)** T cell phenotype stratified by treatment arm. **(D)** Distribution of T cells into CD4^+^, CD8^+^ and CD4^−^CD8^−^ (double negative, DN) populations pre- and post-treatment. (**E-F**) Identification of TCR expression within the T cell populations. Aggregate data from 12 study subjects compared to data from 7 healthy controls for **(E)** αβ and (**F**) γδ respectively. Bars represent medians. *P* values were derived using the Wilcoxon matched pairs test.

Freshly isolated cells were stained and analyzed by flow cytometry to characterize the circulating lymphocyte populations. Data from 30 subjects for which there were adequate numbers of cells for all time points is shown (Fig 1). The distribution of lymphocyte populations, including T cells, B cells, NKT-like and NK cells, was unchanged from pre-treatment through the first ten days of treatment (Fig 1A). At POST we observed a significant decrease in circulating T cells (pre, 73%; post, 63%; p< 0.0001), and a concomitant increase in circulating NK cells (pre, 8%; post, 12%; p = 0.0005). The proportion of B cells was unchanged while NKT-like cells showed a modest yet significant decrease (p = 0.036). Results were not affected by removing the few treatment failures from each group (S1 Fig). The observed changes did not correlate with the severity of disease in terms of lesion size (S2 Fig). Analysis of NK subsets based on CD56 and CD16 markers showed a significant decrease in CD16^+^CD56^+^ cells at POST in the SSG group (Fig 1B).

The subjects were stratified and reanalyzed to determine if the observed changes in cell populations in POST correlated with treatment arm. Similar declines in circulating T cells were seen in both the SSG and TM subjects. Surprisingly, there was no difference when comparing the percentage of T cells in POST between treatment groups (Fig 1C). Similar population changes for NK cells and NKT-like cells were observed in both treatment arms (S3 Fig). We next investigated CD4^+^ and CD8^+^T cells subsets before and after treatment. There was a marked decrease in the median percentage of CD4^+^T cells (pre, 62.3; post, 57.9; *p* = 0.0089) and a proportionate increase in CD8^+^T cells (pre, 30.3; post, 34.8; *p* = 0.0128) post-treatment, with no changes in the CD4^-^CD8^-^ (double negative, DN) population (Fig 1D). We determined the TCR distribution in CL caused by *L*. *major*, using flow cytometry to profile the TCR repertoire of each of the four subsets of T cells (based on CD4 and/or CD8 expression) in our subjects and in healthy donors (n = 7). Here the αβ TCR was exclusively expressed on single-positive CD4^+^T cells and double-positive CD4^+^CD8^+^T cells, and predominantly on the single-positive CD8^+^T cells (representative donor shown, S4 Fig). The DNT cells, on the other hand, were a mixture of αβ expressors, γδ expressors and a population that was negative for both of these TCR. Surprisingly, our results for the αβ and γδ TCR align in healthy and *L*. *major* infected subjects. A decrease in αβ expression (p = 0.052) (Fig 1E) and trend in increase of γδ was observed in POST (Fig 1F) while the overall percentage of DNT cells remained unchanged during the course of the study.

### Both CD4^+^ and CD8^+^T cells play a role in proliferative responses

**Fig 2:**
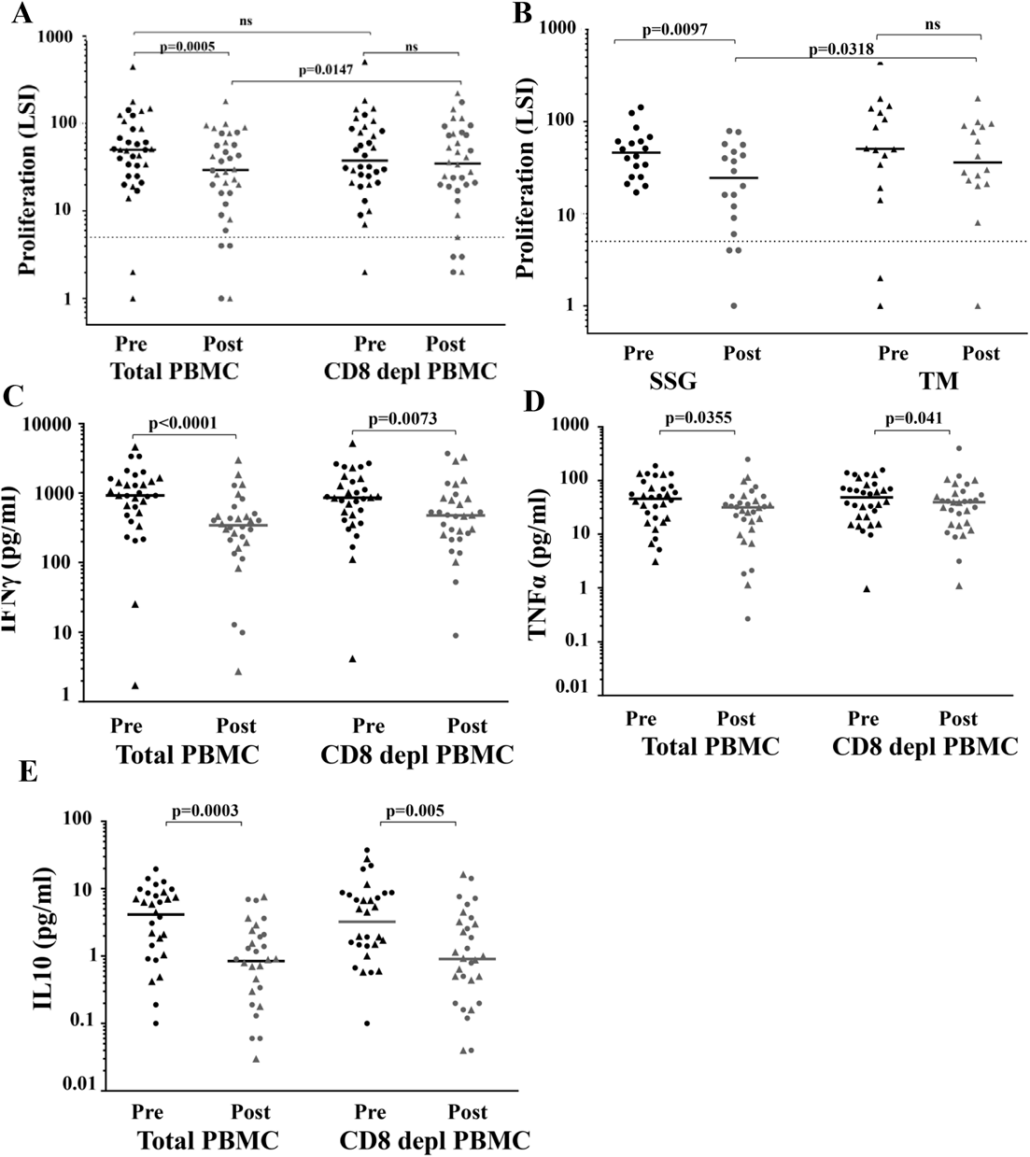
Lymphoproliferative response and cytokine production. (**A**) Whole PBMC or CD8^+^T cell-depleted PBMC (CD8 depl PBMC) from 18 subjects (circles) and 16 subjects (triangles) treated respectively with SSG and TM at pre-treatment (black) and post-treatment (grey) stages were stimulated with SLA for 6 days followed by an 8 hour pulse with [^3^H]-thymidine. Lymphocyte stimulation index (LSI) was determined as fold-increase in mean cpm from triplicate wells over unstimulated wells. An LSI ≥ 5 (dotted line) is considered a positive response. (**B**) *L*. *major* antigen responses in whole PBMC stratified by treatment arm. (**C-E**) Cytokine production following stimulation with SLA. (**C**) IFN-γ, (**D**) TNF-α and (E) IL-10 production by total PBMC or CD8^+^ cell-depleted PBMC was quantified from supernatants sampled on day 6 of the lymphoproliferation assays. Bars represent medians. *P* values were derived using the Wilcoxon matched pairs test.

The lymphoproliferative response in 34 evaluable subjects was analyzed at different time points with concurrent cytokine production. Interestingly, a significant decrease in *Leishmania* antigen-specific T cell proliferation against SLA (p = 0.0005) was seen in POST subjects of total PBMC (Fig 2A). These differences persisted when analyzed without the few treatment failures in each group (S5 Fig). However, when analyzed by treatment arm, this decrease in proliferation after therapy was only observed in the SSG but not TM treatment (Fig 2B).

Recent reports suggest that CD8^+^T cells play a regulatory role in immunity to leishmaniasis [26]. In testing the role of CD8^+^ cells in proliferation responses PRE and POST, we depleted CD8^+^ cells from the bulk PBMC prior to stimulation. The proliferation differences between PRE and POST responses were abrogated with CD8^+^T cell depletion pointing to a potential immunomodulatory or regulatory role for CD8^+^T cells (Fig 2A). Cytokines were quantified to determine if the suppressive effect of the CD8^+^T cells involved soluble mediators. Interestingly, IFN-γ, IL-10 and TNF-α were produced at significantly lower levels in POST, whether the CD8^+^T cells were present or not (Fig 2C, 2D and 2E) which restricts the CD8^+^T cell effects to modulation of lymphocyte proliferation independent of cytokines tested here.

**Fig 3:**
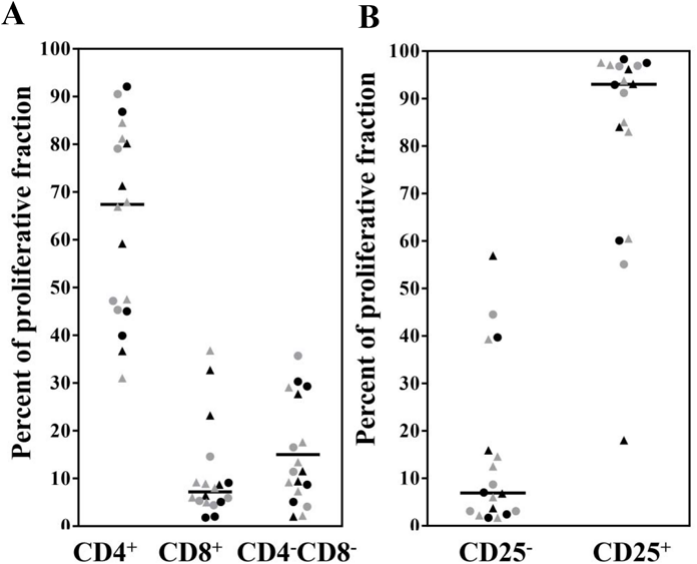
Identification of responding populations by CFDA-SE labeling and flow cytometry analysis. Identification of proliferating lymphocytes based on expression of (**A**) CD4 and CD8 or (**B**) CD25. Circles represent SSG subjects and triangles represent TM subjects. Black and grey are for PRE and POST respectively. Bars represent medians.

We next used CFSE labeling to identify antigen-specific proliferating cell subsets in both bulk and CD8^+^T cell depleted PBMC. Aggregate data is shown in Fig 3Aand 3B and a representative gating example in S6 Fig. While the predominant proliferative fraction consisted of CD4^+^T cells (68%), there was a modest expansion of CD8^+^T cells (7%) and CD4^-^CD8^-^ DNT cells (15%) (Fig 3A). As expected, the vast majority (>90%) of responding cells were activated, as assessed by CD25 expression (Fig 3B).

### Reduced circulating regulatory CD4^+^T cells after healing

**Fig 4:**
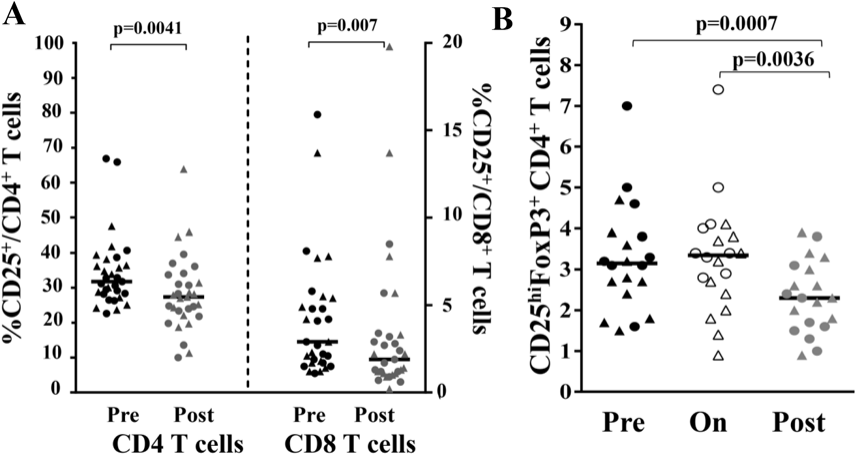
Identification of activated and regulatory T cell populations by flow cytometry. **(A)** Freshly isolated cells were stained for CD3, CD4, CD8 and CD25 for identification of activated T cells. Data obtained from 31 subjects (circles for SSG subjects and triangles for TM subjects) for which pre-treatment (black) and post-treatment (grey) cells were available. Bars represent medians. **(B)** Identification of Treg cells from thawed PBMC. Aggregate data from 20 donors for CD4^+^CD25^high^ Foxp3^+^. P values derived using the Wilcoxon matched pairs test.

Based on CD25 expression and the observed modulation of proliferative immune response, we investigated the role of T regulatory (Treg) cells in the healing process. PBMC were analyzed by flow cytometry to determine the levels of activated T cells, identified by CD25 expression. At POST, we observed a decrease in the percentages of circulating activated T cells in both the CD4 and CD8 compartments (Fig 4A). We identified Treg as those cells within the CD4^**+**^T cell compartment that expressed the highest levels of CD25 (CD25+ bright) and FoxP3 (S7 Fig). Aggregate data from n = 20 sets of subjects shows that while there was no effect on the Treg population during treatment, there was a marked reduction in circulating CD4^**+**^ Treg cells in POST (pre, 3.1%; on, 3.3%; post, 2.3%; p-values = 0.0007 and 0.0036) (Fig 4B).

### Decreased cytokine production in post-treatment polyfunctional CD4^+^ T cells

**Fig 5:**
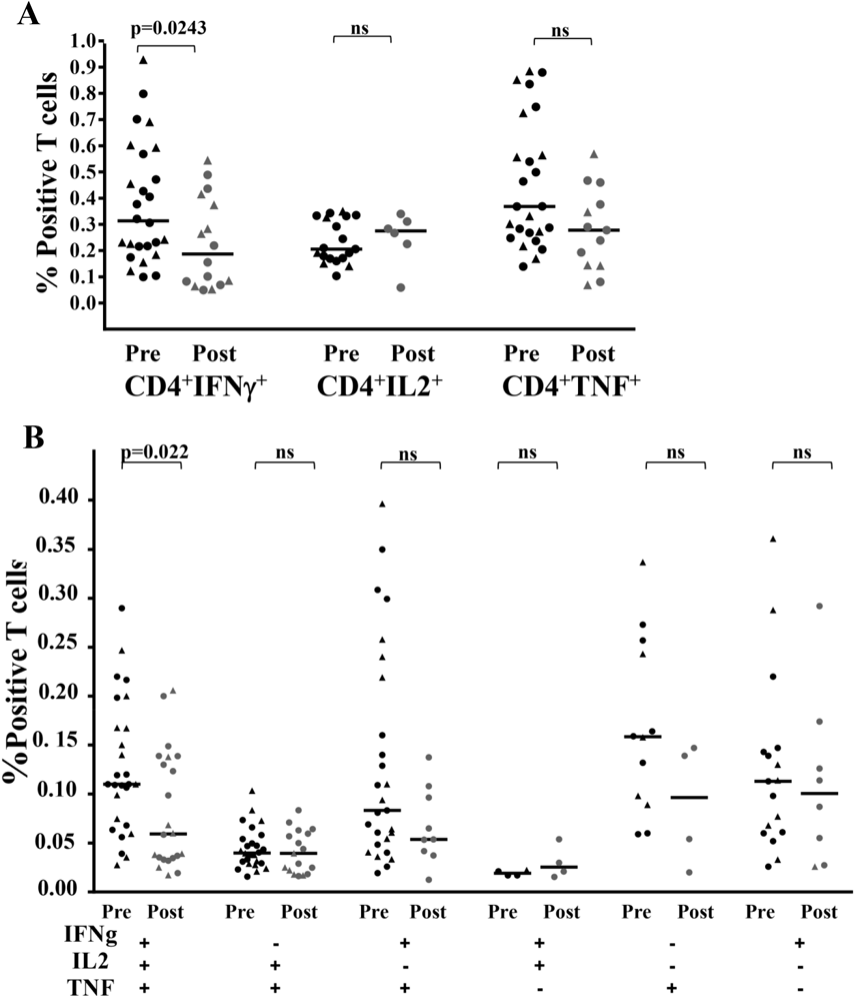
Characterization of the cytokine production capacity of responding T cells. Multiparameter flow cytometry was used to determine (**A**) IFN-γ, IL-2, and TNF-α production in CD4^+^ T cells (B) the frequency of cells expressing each of the seven possible combinations of IFN-γ, IL-2, and TNF-α. Circles represent SSG subjects and triangles represent TM subjects. *P* values derived using the Mann-Whitney test for unpaired samples.

The degree of protection against various infections including leishmaniasis [7] is predicted by the frequency of polyfunctional CD4^+^ memory T cells that produce IFN-γ, TNF-α, and IL-2. We assessed intracellular cytokine production by CD4^+^T cells PRE and POST using multiparameter flow cytometry. First, we were able to independently quantify production of IFN-γ, TNF-α and IL-2 by the CD4^+^ cells, and observed a significant decrease in production of IFN-γ at POST (Fig 5A). Next, we used Boolean gating to analyze the polyfunctionality of these SLA-specific CD4^**+**^T cell responses and found a significant decrease in the frequency of triple positive CD4^**+**^ T cells expressing IFN-γ, IL-2 and TNF-α at POST also (Fig 5B). For the subjects that failed to meet the validation criteria in the Boolean gating (minimum 50 events), no values are reported which explains the fewer numbers of points in certain subsets.

## Discussion

Little is known about the cellular phenotypic profile and immune response of humans prior-to and following treatment with different leishmaniasis therapeutic regimens. In this study, we compared the immune response profile in a cohort of *L*. *major* infected subjects treated with intravenous SSG or locally applied heat therapy (TM) [21]. The mechanism of actions of these two treatment modalities and the nature, location and distribution of therapy are markedly different. Although both treatments resulted in clinical healing, we hypothesized that an immunomodulating systemic therapy might act through different immune mechanisms compared to a localized, physical, direct parasite-killing therapy.

In this study, we report two important findings with functional immunologic underpinnings. First, downmodulation of *Leishmania* antigen-specific CD4^+^T cell proliferative responses possibly through a CD8^+^T-cell dependent mechanism was observed after therapy. Second, we report that *Leishmania*-specific polyfunctional CD4^+^T cells also decrease after therapy.

Since clinical cure from leishmaniasis is classically and primarily dependent on T cell subtypes and relevant cytokine production profiles [27,28], cells were phenotyped from subjects before and after treatment. After treatment and independent of the treatment modality, circulating T cells and NKT-like cells were decreased with a concomitant increase in circulating NK cells highlighting the relevance of the innate immune system for *Leishmania* control. NK and T cells seemed to have reciprocal effects; wherein NK cell-produced IFN-γ which resulted in T cell activation and the T cell derived IL-2 lead to NK triggering [29]. Similarly, an association between the increased frequency of NK cells and lesion healing is reported after immunotherapy with BCG/*Leishmania* antigens [30].

NKT-like cells share several characteristics with NK cells [31] and serve as frontline innate immune effectors and potential regulators of adaptive immune responses against microorganisms [32]. Although only a trend, the increase of NKT-like cells observed during treatment could be explained by their ability to serve as an early source of regulatory cytokines and their degranulation-related killing function.

In our T cell subset analysis, we showed a high percentage of CD4^+^T cells in the early treatment phase, suggesting their association with disease progression [33]; while the percentage of CD8^+^T cells increased post treatment. This could reflect the down-modulation of the immune response, as a means to mitigate immunopathology, consistent with other studies linking CD8^+^T cell subset induction with the healing process [26] and lesion resolution during antimonial therapy [34]. Contraction of CD4^+^T cells and expansion of CD8^+^T cells during healing suggests CD4 modulation after cure [35]. CD8^+^T cells were also increased in healed Brazilian CL subjects suggesting potential modulation of the activity of CD4^+^ cells by direct cytolytic effect of infected macrophages, or by other regulatory effects [33]. Our results confirm that a balance between the proportion of CD4^+^ and CD8^+^T cells is important for leishmaniasis healing [33,36–38]. We also analyzed DN T cells, and in particular the αβ subpopulation, a highly activated T cell subset producing cytokines to activate monocytes and macrophages [39]. DN lymphocytes are the second most prevalent cell type producing IFN-γ in human CL [40] and contribute to a leishmanicidal immune environment [39]. DN T cells were recently described as important players in effective and protective primary and secondary anti *L*. *major* immunity in experimental cutaneous leishmaniasis [41]. Leishmania-reactive DN T cells express predominantly αβ TCR, are restricted by MHC class II molecules, lack immunoregulatory properties and display transcriptional profile distinct from conventional CD4+ T cells. Current dogma that DN T cells are CD4 and CD8 T cells that have lost their co-receptors is being challenged by the emerging theory that Fas-mediated apoptosis actively removes normally existing DN T cells from the periphery. Impaired Fas-mediated apoptosis may lead to accumulation of these cells rather than *de novo* generation of DN T cells from activated CD4 or CD8 T cells [42].

In our study, both αβ and γδ subpopulations were similarly represented in the *L*. *major* and uninfected control subjects and remained stable during the course of treatment. DN T cell population changes were previously described in human infection with *L*.*(V) braziliensis*. In that study, 75% of DN T cells from subjects expressed the αβ TCR compared to uninfected persons where 80% of DN T cells express the γδ TCR [39]. This discordance was not observed here and this may be attributed to different *Leishmania* species with differing disease patterns and/or genetic backgrounds of the individuals studied.

*Leishmania* induced immunity is based upon the generation of memory T cells that recognize cognate *Leishmania* antigens and proliferate after exposure thus activating the effector cells [43]. In our study, responses to SLA were consistently diminished in the post treatment phase. Surprisingly, the proliferative responses were significantly decreased only for subjects receiving systemic treatment but not subjects receiving local treatment. This could in part be explained by the higher numbers of treatment failures at 6 months in TM (4/19 in TM group versus 2/20 in SSG group) causing LPA due to parasite persistence. Similarly to our findings, others also report a decline of the lymphoproliferative response after therapy [28,36–38].

The CD8^+^T cell-dependent decrease in CD4^+^T cell proliferation suggested a post treatment, curative type counter-regulatory mechanism. In contrast, in a BALB/c mouse model, CD8 T cell depletion did not interfere with the proliferative ability of draining lymph node CD4 T cells and was associated with an increase in parasite load [44]. As demonstrated for CD4 T cells [45], CD8 immunomodulation maybe due, for example, to up- regulation of Fas expression on CD4 to induce their apoptotic death. We know that CD8 T cells play a role in the healing process and resistance to reinfection in New World human CL. Conversely, other studies associate CD8^+^ to tissue injury [46]. Recently, it was hypothesized that changes in the frequency of effector CD8^+^ T cells, during and after antimonial therapy is a critical step to generate an efficient immune response either for by triggering or resolving the lesion [34]. *In vivo* experiments with human cells showed that CD8 T cells produce IFN-γ and drive Th1 differentiation [47]. However in our study, after treatment, all subjects showed decreased IFN-γ, IL-10 and TNF-α levels, with or without CD8^+^ depletion. This indicated that CD8^+^T cell mediated regulation of the CD4^+^T cell response was not attributable to the soluble mediators studied here. The high IFN-γ production observed pre-treatment suggests that the subjects have initiated an immune response to eliminate the parasite [48]. Additionally, during effective treatment, gradual parasite destruction by macrophages is expected with a diminishing parasite load. Overall, our results add evidence that local heat therapy of CL elicits a systemic cytokine response similar to that of systemic pentavalent antimony. In fact, a decrease in IFN-γ, IL-5 and TNF-α in both groups was seen at day 28 post treatment with meglumine antimoniate in a previous study [49]. These results indicate that proinflammatory responses were progressively downmodulated after therapy and that the cytokine profile produced after cure is shaped during the active phase of disease [50].

Our results were contrary to our hypothesis, as the subjects in the both treatment arms generally exhibited similar cellular immune response profiles. This may be explained, in part, by the tendency of CL to eventually self-heal so cure processes may have occurred despite therapy [51]. Another potential limitation of our study is that there were fewer subjects collected at the 6 month time point, however this was similar between treatment arms. A local immune analysis in the skin may have provided additional clues to immune response alterations induced by different treatments, as might an earlier post timepoint.

Taken together, our findings highlight the existence of regulatory mechanisms that counterbalance early immune responses without altering the CL healing outcome. The magnitude of effector T cell responses can be controlled by regulatory T cells at the lesion site by suppressing lymphocyte proliferation [52]. These mechanisms are important to maintain the host tissue integrity against a subsequent or persistent inflammatory response. Induction of Tregs during chronic infections results from antigen presentation in a particular cytokine environment [53,54]. Interestingly, we found that the percentage of CD25^hi^CD4^+^Foxp3^+^ cells decreased after treatment suggesting that Tregs may be responsible for the suppression that was associated with healing and that their drop is not an artifact of CD4 decrease demonstrated earlier. Tregs have been shown to substantially contribute to tissue repair by providing regulation at sites of healing [55].

To gain a better understanding of the complex immunopathogenesis of CL, study of the quality of a Th1 response, not solely its magnitude, was recently adopted [7,8]. Our analysis evaluated polyfunctional CD4^+^T cells in response to treatment. Overall, we observed a contraction in polyfunctional CD4^+^T cells in the post-treatment group, both in terms of number of responding cells and production of multiple cytokines.

In conclusion, healing of CL is a dynamic but consistent process. Similar changes in frequency, quality, and antigen specific responses were observed in both treatment arms and may represent a signature for curative responses.

## Supporting Information

S1 Fig Characterization of lymphocyte populations by flow cytometry in completely healed subjects (removing the subjects who failed to cure their lesions at 6 months). Data is presented from 27 subjects (15 in the SSG arm and 12 in TM arm represented in circles and triangles respectively) for which cells from all three time points were available. Percentage of lymphocytes positive for surface expression of CD3 (T cells), CD19 (B cells), CD16/CD56 (CD3^-^: NK cells; CD3^+^: NKT-like cells).
(TIF)

S2 Fig Characterization of lymphocyte populations by flow cytometry in healed patients. Data is presented from 30 subjects (17 in the SSG arm and 13 in TM arm represented in circles and triangles respectively, red represents subjects for whom lesion size was above 1000mm) for which cells from all three time points were available. Percentage of lymphocytes positive for surface expression of CD3 (T cells), CD19 (B cells), CD16/CD56 (CD3^-^: NK cells; CD3^+^: NKT-like cells).
(TIF)

S3 Fig NK and NKT-like subpopulations stratified by treatment arm. (TIF)

S4 Fig Identification of TCR expression within the T cell populations. Representative donor showing flow cytometry analysis.
(TIF)

S5 Fig Lymphoproliferative response and cytokine production in completely healed subjects (removing the subjects who failed to cure their lesions at 6 months). Whole PBMC or CD8^+^T cell-depleted PBMC (CD8 depl PBMC) from 16 subjects (circles) and 14 subjects (triangles) treated respectively with SSG and TM at pre-treatment (black) and post-treatment (grey) stages were stimulated with SLA for 6 days followed by an 8 hour pulse with [^3^H]-thymidine. Lymphocyte stimulation index (LSI) was determined as fold-increase in mean cpm from triplicate wells over unstimulated wells. An LSI ≥ 5 (dotted line) is considered a positive response.
(TIF)

S6 Fig Identification of responding populations by CFDA-SE labeling and flow cytometry analysis. Representative sample showing gating strategy and identification of the proliferative fraction of lymphocytes.
(TIF)

S7 Fig Identification of Treg cells from thawed PBMC. Gating example showing Treg cells were identified as viable lymphocytes positive for CD3 and CD4 expressing high levels of CD25 and positive for the transcription factor FoxP3.
(TIF)
