# Supplementary figures and images for: The Immunology of a Healing Response in Cutaneous Leishmaniasis Treated with Localized Heat or Systemic Antimonial Therapy
Source: PLoS Negl Trop Dis. 2015 Oct 20;9(10):e0004178. doi: 10.1371/journal.pntd.0004178 (PMC4618688; doi:10.1371/journal.pntd.0004178)

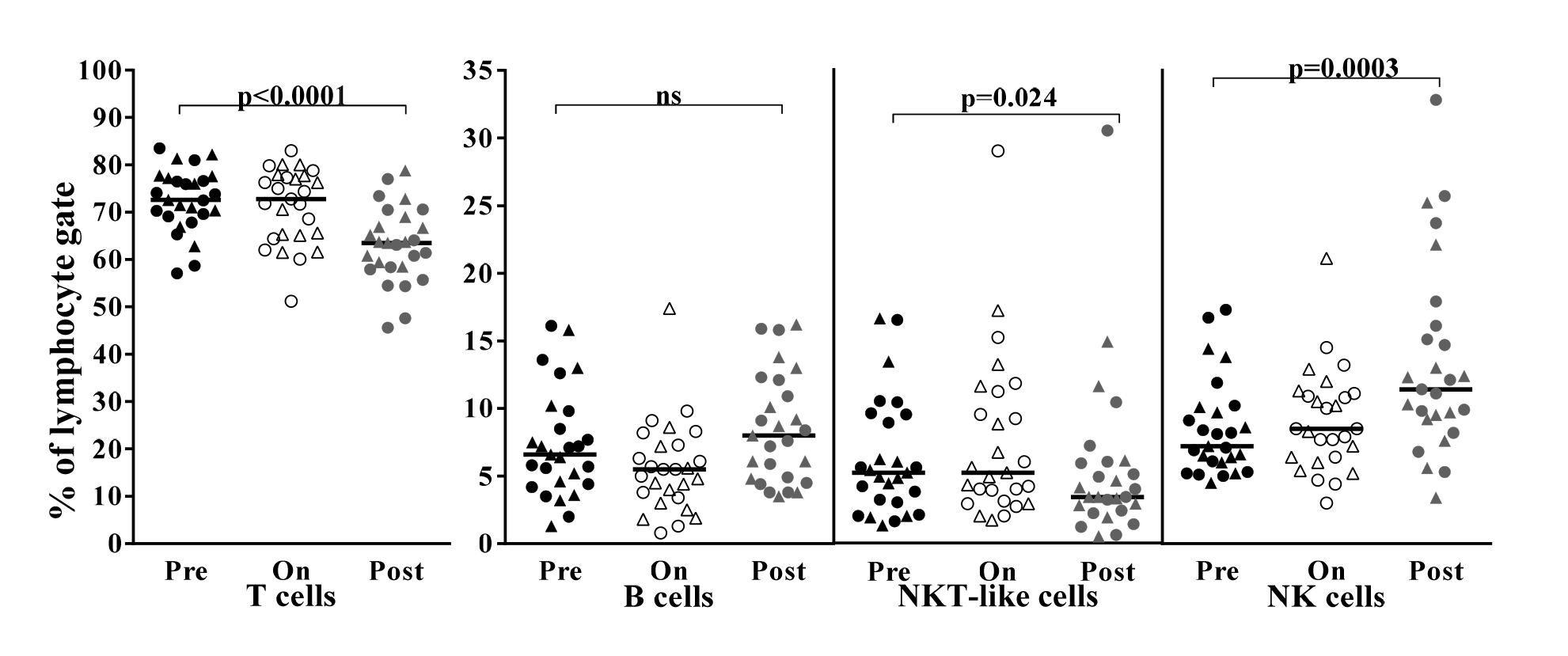

Supplement: S1 Fig — Data is presented from 27 subjects (15 in the SSG arm and 12 in TM arm represented in circles and triangles respectively) for which cells from all three time points were available. Percentage of lymphocytes positive for surface expression of CD3 (T cells), CD19 (B cells), CD16/CD56 (CD3-: NK cells; CD3+: NKT-like cells). (TIF) [file pntd.0004178.s001.tif]

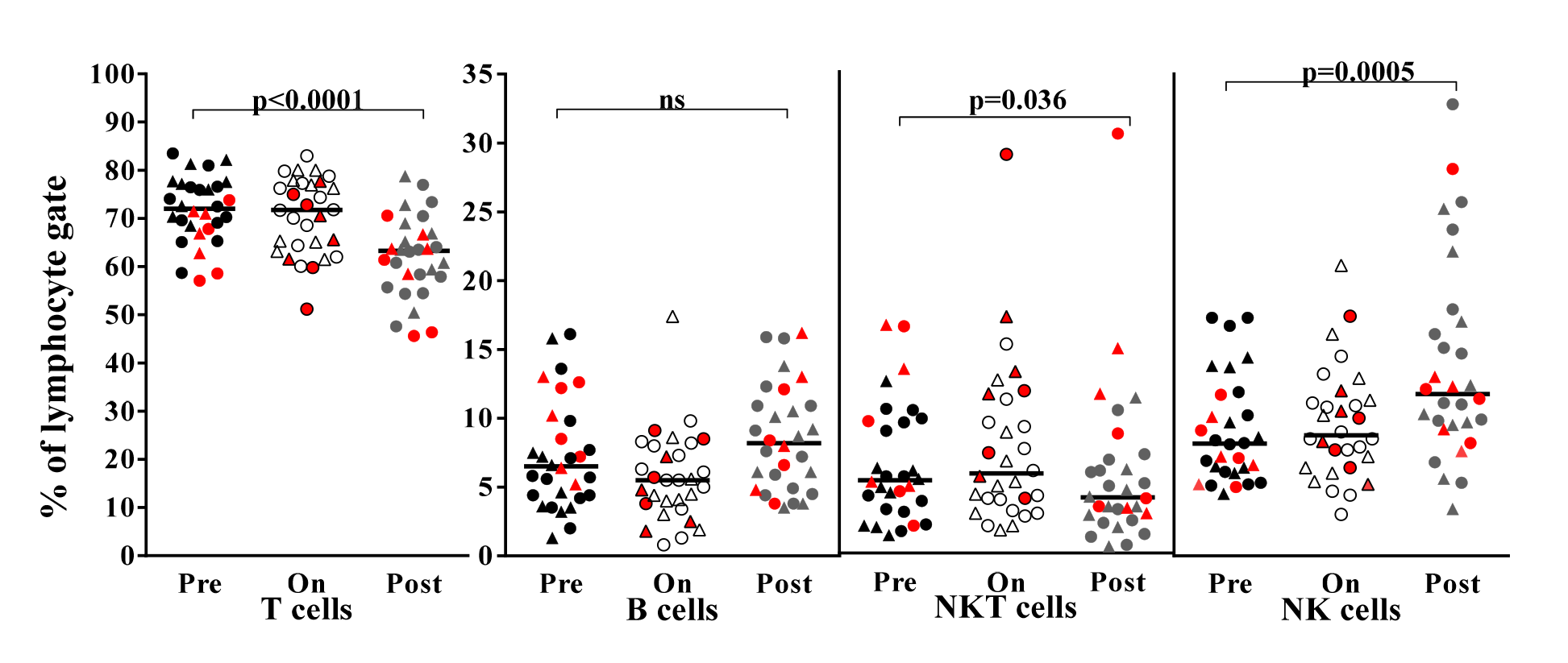

Supplement: S2 Fig — Data is presented from 30 subjects (17 in the SSG arm and 13 in TM arm represented in circles and triangles respectively, red represents subjects for whom lesion size was above 1000mm) for which cells from all three time points were available. Percentage of lymphocytes positive for surface expression of CD3 (T cells), CD19 (B cells), CD16/CD56 (CD3-: NK cells; CD3+: NKT-like cells). (TIF) [file pntd.0004178.s002.tif]

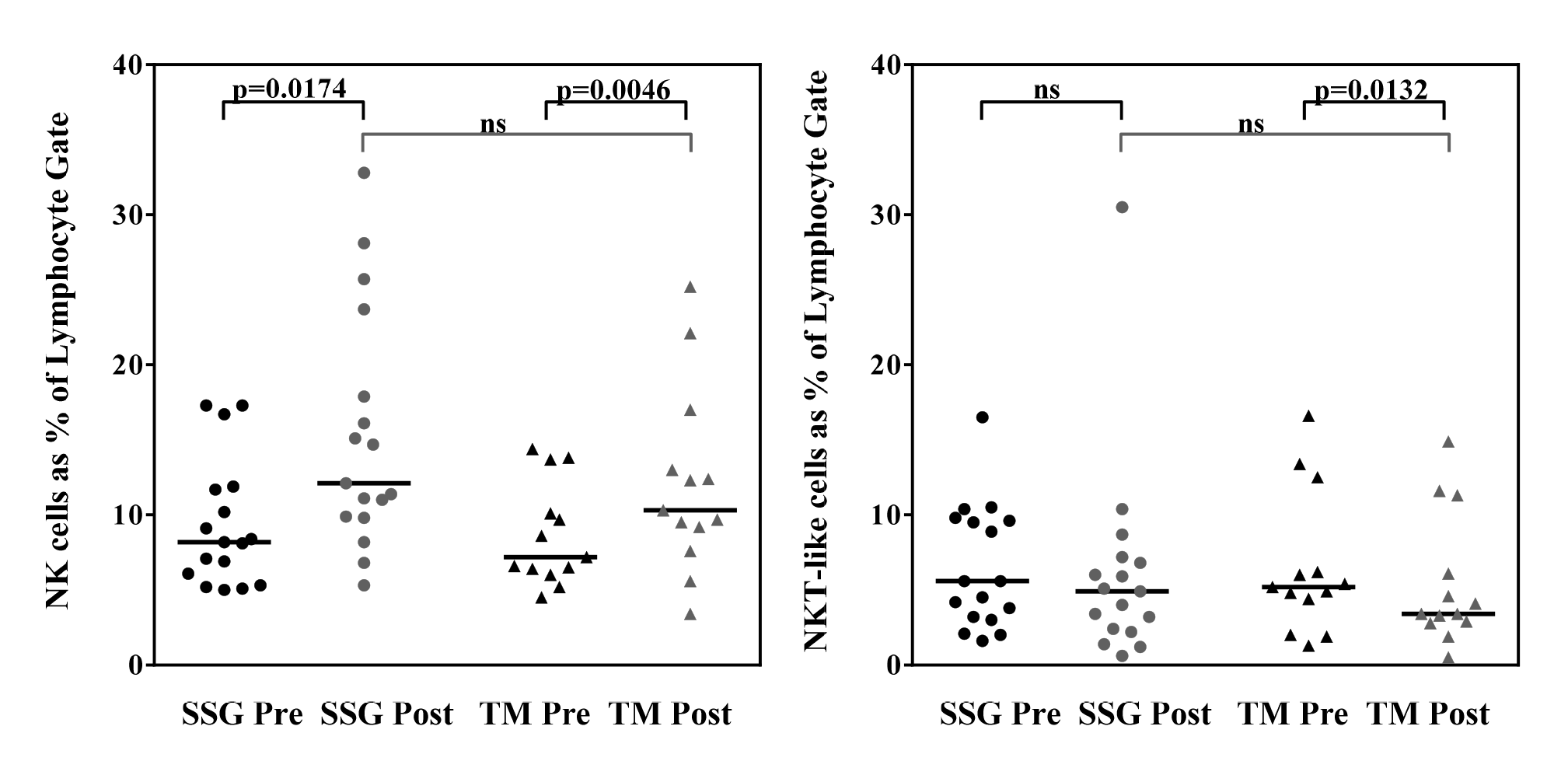

Supplement: S3 Fig — (TIF) [file pntd.0004178.s003.tif]

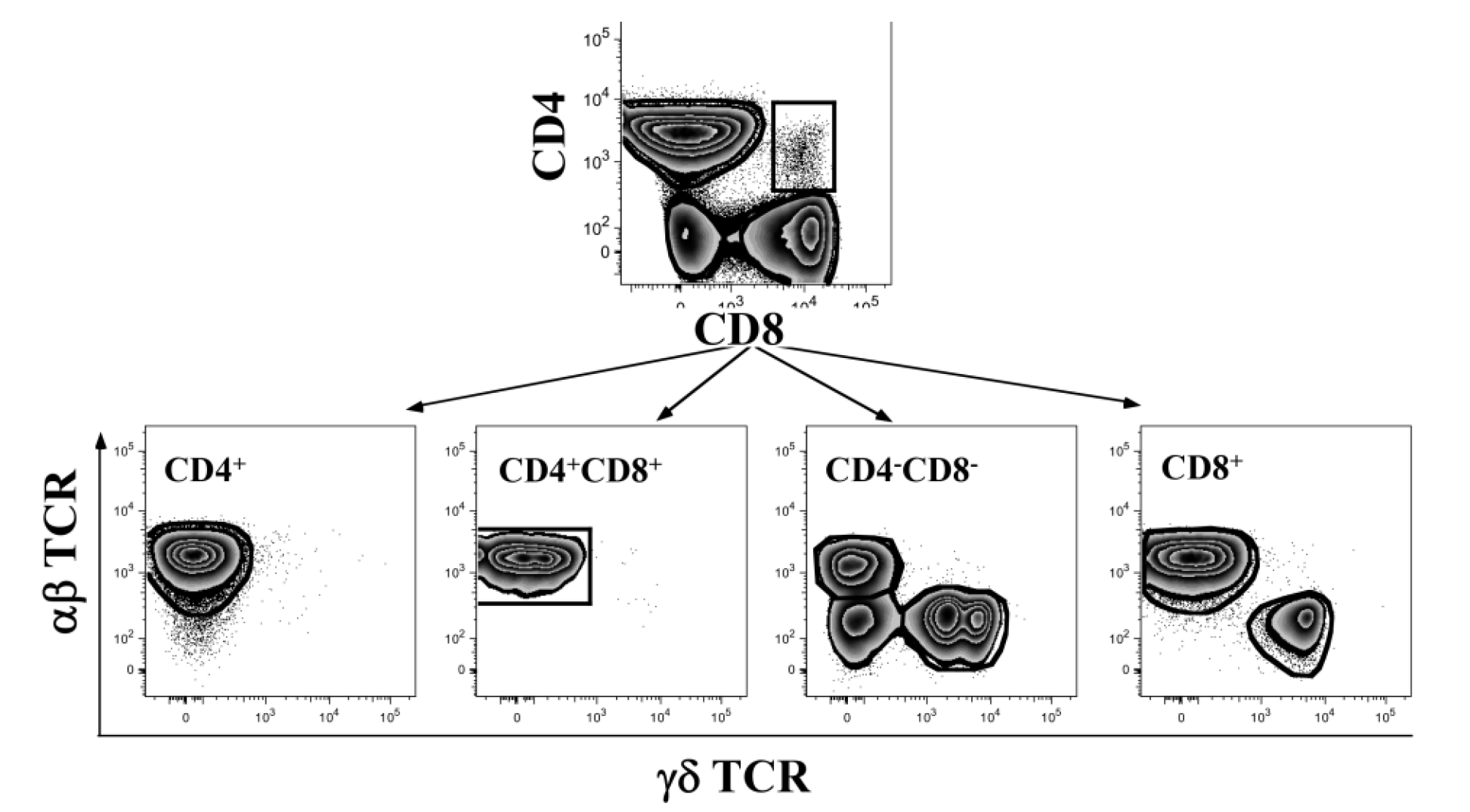

Supplement: S4 Fig — Representative donor showing flow cytometry analysis. (TIF) [file pntd.0004178.s004.tif]

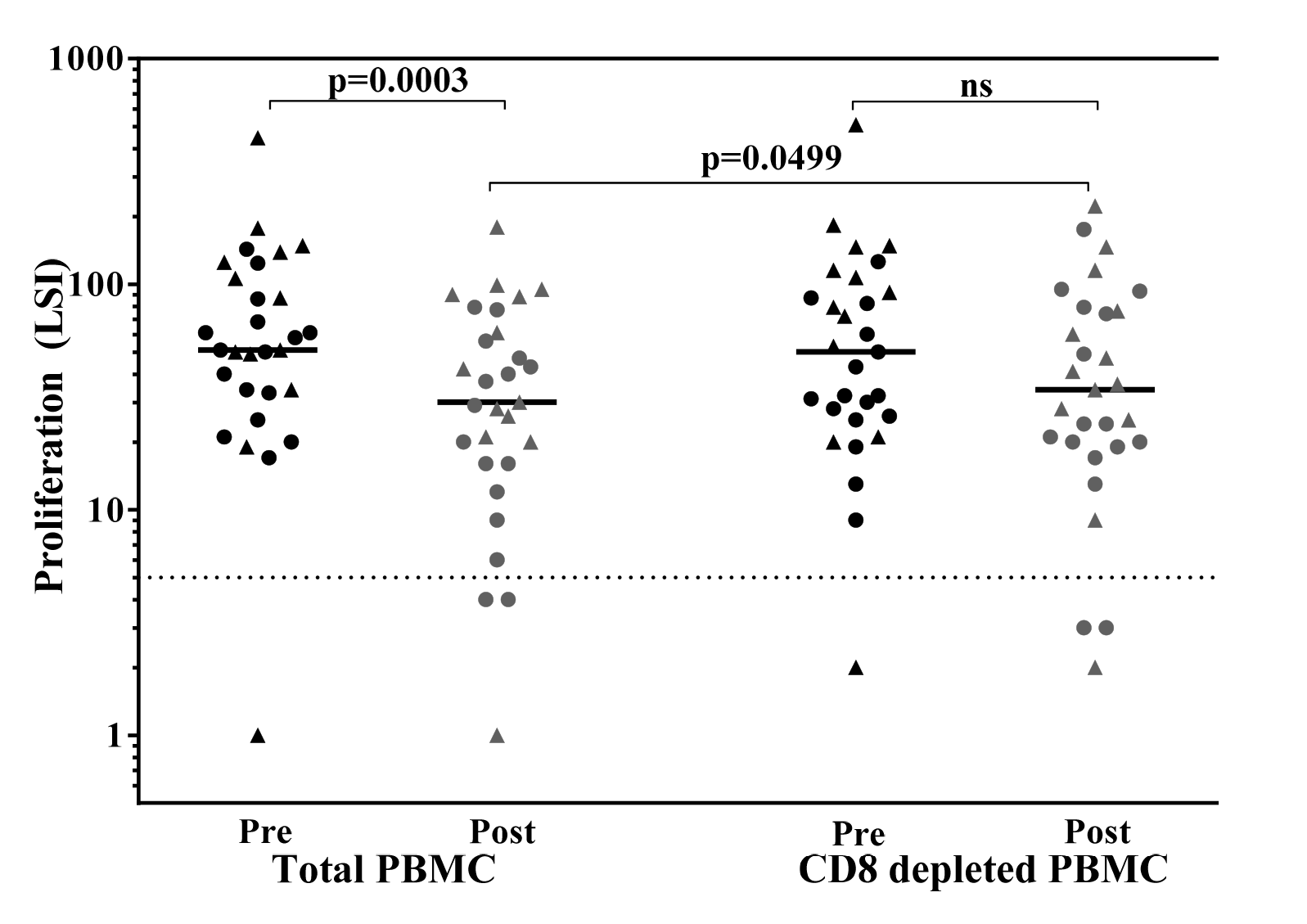

Supplement: S5 Fig — Whole PBMC or CD8+T cell-depleted PBMC (CD8 depl PBMC) from 16 subjects (circles) and 14 subjects (triangles) treated respectively with SSG and TM at pre-treatment (black) and post-treatment (grey) stages were stimulated with SLA for 6 days followed by an 8 hour pulse with [3H]-thymidine. Lymphocyte stimulation index (LSI) was determined as fold-increase in mean cpm from triplicate wells over unstimulated wells. An LSI ≥ 5 (dotted line) is considered a positive response. (TIF) [file pntd.0004178.s005.tif]

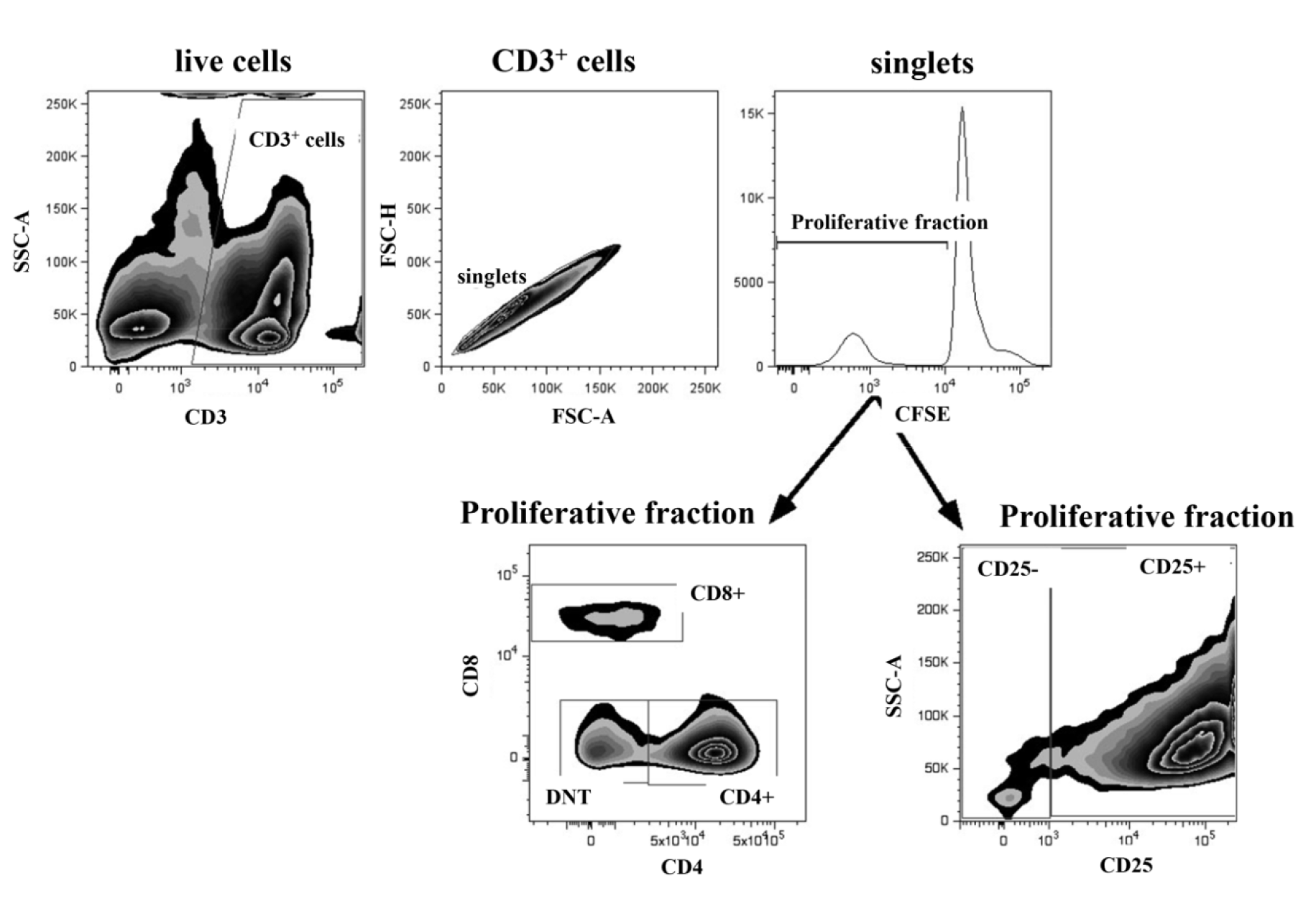

Supplement: S6 Fig — Representative sample showing gating strategy and identification of the proliferative fraction of lymphocytes. (TIF) [file pntd.0004178.s006.tif]

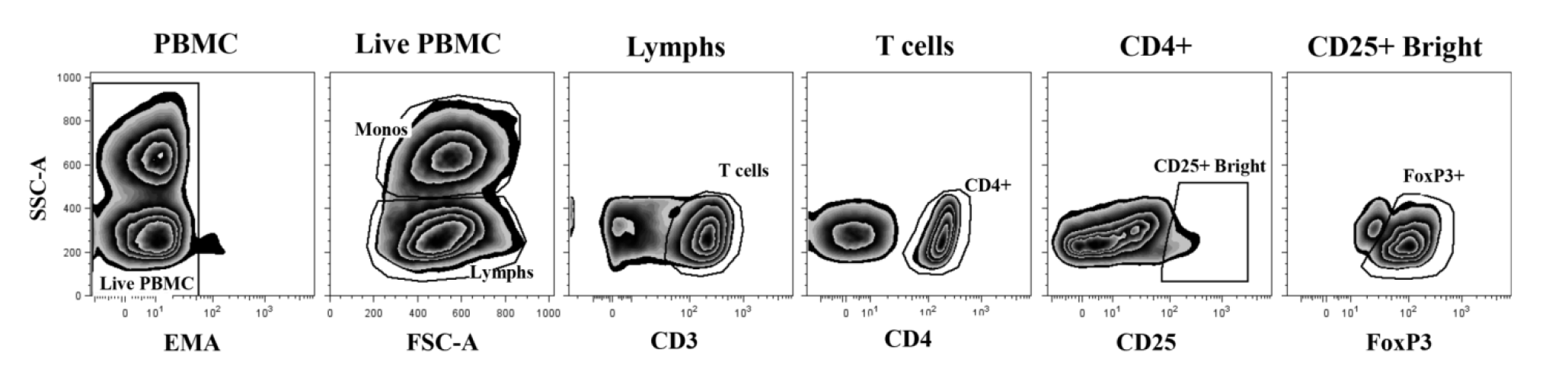

Supplement: S7 Fig — Gating example showing Treg cells were identified as viable lymphocytes positive for CD3 and CD4 expressing high levels of CD25 and positive for the transcription factor FoxP3. (TIF) [file pntd.0004178.s007.tif]
